# Long-term functional outcomes in patients with isolated cerebellar infarction: the KOSCO study

**DOI:** 10.3389/fneur.2025.1541245

**Published:** 2025-03-10

**Authors:** Ho Seok Lee, Min Kyun Sohn, Jongmin Lee, Deog Young Kim, Yong-Il Shin, Gyung-Jae Oh, Yang-Soo Lee, Min Cheol Joo, So Young Lee, Min-Keun Song, Junhee Han, Jeonghoon Ahn, Young-Hoon Lee, Dae Hyun Kim, Young-Taek Kim, Yun-Hee Kim, Won Hyuk Chang

**Affiliations:** ^1^Department of Physical and Rehabilitation Medicine, Center for Prevention and Rehabilitation, Heart Vascular Stroke Institute, Samsung Medical Center, Sungkyunkwan University School of Medicine, Seoul, Republic of Korea; ^2^Department of Rehabilitation Medicine, College of Medicine, Chungnam National University, Daejeon, Republic of Korea; ^3^Department of Rehabilitation Medicine, Konkuk University School of Medicine, Seoul, Republic of Korea; ^4^Department and Research Institute of Rehabilitation Medicine, Yonsei University College of Medicine, Seoul, Republic of Korea; ^5^Department of Rehabilitation Medicine, Pusan National University School of Medicine, Pusan National University Yangsan Hospital, Yangsan, Republic of Korea; ^6^Department of Preventive Medicine, Wonkwang University, School of Medicine, Iksan, Republic of Korea; ^7^Department of Rehabilitation Medicine, School of Medicine, Kyungpook National University, Kyungpook National University Hospital, Daegu, Republic of Korea; ^8^Department of Rehabilitation Medicine, Wonkwang University School of Medicine, Iksan, Republic of Korea; ^9^Department of Rehabilitation Medicine, Jeju National University Hospital, Jeju National University School of Medicine, Jeju City, Republic of Korea; ^10^Department of Physical and Rehabilitation Medicine, Chonnam National University Medical School, Gwangju, Republic of Korea; ^11^Department of Statistics, Hallym University, Chuncheon, Republic of Korea; ^12^Department of Health Convergence, Ewha Womans University, Seoul, Republic of Korea; ^13^Department of Preventive Medicine, Chungnam National University Hospital, Daejeon, Republic of Korea; ^14^Department of Physical and Rehabilitation Medicine, Sungkyunkwan University School of Medicine, Suwon, Republic of Korea; ^15^Department of Health Science and Technology, Department of Medical Device Management and Research, SAIHST, Sungkyunkwan University, Seoul, Republic of Korea

**Keywords:** ischemic stroke, cerebellum, long-term outcome, functional prognosis, recovery

## Abstract

**Background:**

There are relatively few reports on the long-term sequential functional recovery and prognosis in patients with cerebellar infarction. The aim of this study was to investigate the long-term recovery of multifaceted functional outcomes up to 36 months after onset and the functional prognosis of isolated cerebellar infarction.

**Methods:**

This study was a retrospective analysis of the Korean Stroke Cohort for Functioning and Rehabilitation (KOSCO) data up to 36 months after onset. Isolated cerebellar infarction was defined as the presence of lesions in the cerebellum without lesions in other brain parenchyma. We assessed multifaceted functional domains, including motor (Fugl-Meyer Assessment, FMA), ambulatory (Functional Ambulation Category, FAC), cognitive (Korean Mini-Mental State Examination, K-MMSE), swallowing (American Speech-Language-Hearing Association National Outcome Measurement System Swallowing Scale, ASHA-NOMS), and language functions (Short version of the Korean Frenchay Aphasia Screening Test, Short K-FAST), using serial measurements. In addition, functional outcome was assessed with the Functional Independence Measure (FIM) up to 36 months after onset.

**Results:**

Among 390 screened isolated cerebellar infarction patients, a total of 183 patients were included in this study. Cognitive (mean[SD] of K-MMSE 27.6 ± 3.6) and swallowing (ASHA-NOMS 6.8 ± 0.7) functions showed significant improvement up to 3 months (*p* < 0.05). Motor (FMA 98.8 ± 3.8) and language (ASHA-NOMS 6.9 ± 0.4) functions improved significantly up to 6 months (*p* < 0.05). Furthermore, ambulatory function (FAC 4.7 ± 0.9) and functional independency (FIM 122.2 ± 12.0) continued to improve up to 12 months (*p* < 0.05). Vascular territory involving superior cerebellar artery, older age, female sex, and greater initial severity were identified as negative independent prognostic factors predicting functional outcome measured by FIM at 12 months after stroke.

**Conclusion:**

The plateau of recovery in multifaceted functional outcomes varied among patients with cerebellar infarction. Functional independence plateaued at 12 months and showed a relatively favorable prognosis up to 36 months after stroke.

## Introduction

Cerebellar infarction accounts for a relatively small proportion of all strokes. Previous studies have reported that the incidence of cerebellar infarction accounts for approximately 2 ~ 3% of all ischemic stroke (1–3). The pathophysiology and clinical presentation of cerebellar infarction are diverse and well known from previous studies (1–6). Among the vascular territories supplying the cerebellum, the posterior inferior cerebellar artery (PICA) is the most commonly affected, followed by the superior cerebellar artery (SCA) and the anterior inferior cerebellar artery (AICA) (2, 5). Cardioembolism and large-vessel atherosclerosis are the most common causes of cerebellar infarction (6).

Patients with cerebellar infarction typically present with a range of symptoms, including dysarthria, ataxia, gait disturbance, nystagmus, or altered mental status. Consequently, previous studies have reported the functional outcomes of patients with cerebellar infarction using various assessments, such as the Functional Independence Measure (FIM), the modified International Cooperative Ataxia Rating Scale (MICARS), the National Institutes of Health Stroke Scale (NIHSS), and the modified Rankin Scale (mRS) (7–10). However, these studies with relatively small sample sizes of participants did not comprehensively assess multifaceted functional outcomes. Furthermore, there is a lack of research on the long-term, sequential patterns of functional recovery and prognosis in patients with cerebellar infarction. Additionally, while previous studies have identified older age as a factor influencing outcomes in stroke patients, including those with cerebellar infarction, there is a lack of detailed reports on the distinct trajectories of functional recovery among younger and older patients with cerebellar infarction (11–13).

Therefore, the primary aim of this study was to investigate the long-term recovery patterns of multifaceted functional outcomes up to 36 months after onset and the functional prognosis of isolated cerebellar infarction. The secondary aim was to analyze differences in functional recovery patterns between young and old cerebellar infarction patients.

## Methods

### Data collection

The Korean Stroke Cohort for Functioning and Rehabilitation (KOSCO) is a prospective, multi-center cohort study of patients with first-time stroke. Data were collected from 9 different hospitals in Korea (14). All patients provided written informed consent, and the study protocol was approved by the institutional review board of the participating hospitals (details provided in Supplementary Table S1).

This study was a retrospective analysis of the KOSCO data up to 36 months after onset. The affected side of the lesion, vascular territories, and etiology of each patient based on the TOAST classification (15) were documented. Clinical characteristics including demographic information (age, sex, body mass index [BMI], smoking and alcohol history), risk factors for stroke such as hypertension, diabetes, coronary artery disease, atrial fibrillation, and hyperlipidemia recorded during admission, comorbidities, and pre-stroke functional level, were documented (16). Charlson’s weighted comorbidity index (WIC) was used to assess comorbidities (17). The mRS score was used to assess pre-stroke functional level (18). The NIHSS score recorded at initial hospitalization was used to assess initial stroke severity (19). The duration of 1st hospitalization, records of whether the patients received inpatient rehabilitation therapy, and information regarding medical complications during the 1st hospitalization were recorded. These complications included thromboembolic disease, pneumonia, urinary tract infection, fall and injuries.

The following functional assessments for multiple domains were performed at 7 days and 3, 6, 12, 18, 24, 30, and 36 months after stroke onset; Fugl-Meyer Assessment (FMA; range 0–100) (20) for motor function, Functional Ambulatory Category (FAC; range 0–5) (21) for ambulatory function, Korean Mini-Mental State Examination (K-MMSE; range 0–30) (22) for cognitive function, American Speech-Language-Hearing Association National Outcome Measurement System Swallowing Scale (ASHA-NOMS; range 0–7) (23) for swallowing function, and the short version of the Korean Frenchay Aphasia Screening Test (short K-FAST; range 0–20) (24) for language function. Functional independence, assessed by the FIM (range 18–126), was measured at 3 to 36 months after onset (25, 26).

### Selection of KOSCO participant for isolated cerebellar infarction

**Figure 1:**
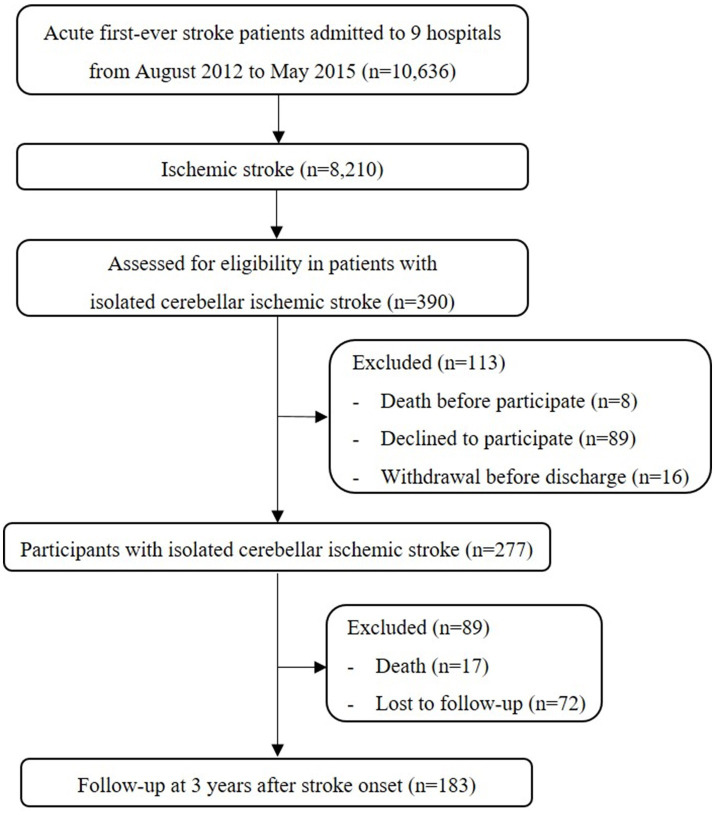
Summary of study participants.

Between August 2012 and May 2015, a total of 8,210 patients with first-time ischemic stroke were screened from 9 different hospitals in Korea. In this study, isolated cerebellar infarction was defined as follows: (1) the presence of ischemic lesions exclusively in the cerebellum, as determined by brain MRI or CT scans. Lesions in the brainstem were explicitly excluded. (2) The identification of the affected cerebellar vascular territories. According to this definition, 390 (4.8%) out of the 8,210 ischemic stroke patients had an isolated cerebellar lesion. Eight patients (2.1%) died before deciding to participate, and 105 patients (26.9%) refused or withdrew from participation in the KOSCO. Of the remaining 277 patients, 17 (6.1%) died, and 72 (26.0%) were lost to follow-up (baseline characteristic details of excluded patients are provided in Supplementary Table S2). Finally, a total of 183 patients with isolated cerebellar infarction were included in this analysis (Figure 1).

### Statistical analysis

Categorical variables are presented as numbers of frequencies and percentages or median and interquartile range (IQR). Numerical variables are summarized as means and standard deviations (SD). To classify subgroups according to age, we categorized patients younger than 65 as young patient group and those 65 or older as old patient group (27, 28). Differences in demographic and clinical characteristics of stroke patients between young and old patients were analyzed using independent t-test and chi-square test for numerical and categorical variables, respectively. Paired t-test and Wilcoxon signed-rank test, with Bonferroni correction, were used to analyze differences between the time points from stroke onset for functional outcomes of multiple domains for numerical and ordinal variables, respectively. To evaluate prognostic factors for functional independence, multivariable logistic regression analysis was performed with factors that were found to be statistically significant in univariable regression analysis.

The *p*-value for statistical significance was set at <0.05 for all analyses performed in this study. Statistical analyses were performed with SPSS version 25.

## Results

### Patient characteristics

The results of the demographic and clinical characteristics of the participants are shown in Table 1. The mean (SD) age of patients was 61.0 ± 11.8 years, and 138 (75.4%) were male. The most common vascular territory involved was PICA (80.3%). According to TOAST classification, large artery atherosclerosis (38.3%) was the most common etiology, followed by small vessel occlusion (25.1%). Among the risk factors, hypertension was the most common risk factor in 103 patients (56.3%). The median (IQR) premorbid mRS score of the patients was 0 (0–1). The initial and baseline severity as measured by the median (IQR) NIHSS score were 0 (0–2) and 0 (0–1), respectively.

**Table 1:** Demographic and clinical characteristics of the participants.

Demographic and clinical characteristics	No.(%), mean (SD), or median (IQR)			*p*-value
	Total (*n* = 183)	Young (<65) (*n* = 111)	Old (≥65) (*n* = 72)	
Vascular territory				0.64
Superior cerebellar artery	22 (12.0%)	12 (10.8%)	10 (13.9%)	
Anterior inferior cerebellar artery	7 (3.8%)	4 (3.6%)	3 (4.2%)	
Posterior inferior cerebellar artery	147 (80.3%)	90 (81.1%)	57 (79.2%)	
Multiple involvement	7 (3.8%)	5 (4.5%)	2 (2.8%)	
Etiology				0.06
Large artery artherosclerosis	70 (38.3%)	36 (32.4%)	34 (47.2%)	
Small vessel occlusion	46 (25.1%)	31 (27.9%)	15 (20.8%)	
Cardioembolism	25 (13.7%)	12 (10.8%)	13 (18.3%)	
Other determined	20 (10.9%)	15 (13.5%)	5 (6.9%)	
Undetermined	22 (12.0%)	17 (15.3%)	5 (6.9%)	
Age	61.0 ± 11.8	53.3 ± 8.0	72.7 ± 5.2	<0.001^**^
Sex, male	138 (75.4%)	87 (78.4%)	51 (70.8%)	0.29
Body mass index	24.0 ± 3.1	24.2 ± 3.0	23.9 ± 3.2	0.54
Smoking, current	59 (32.2%)	50 (45.0%)	9 (12.5%)	<0.001^**^
Alcohol, current	97 (53.0%)	67 (60.4%)	30 (41.7%)	0.02^*^
Risk factors				
HTN	103 (56.3%)	58 (52.3%)	45 (62.5%)	0.22
DM	40 (21.9%)	19 (17.1%)	21 (29.2%)	0.07
Coronary heart disease	13 (7.1%)	5 (4.5%)	8 (11.1%)	0.14
Atrial fibrillation	16 (8.7%)	6 (5.4%)	10 (13.9%)	0.06
Hyperlipidemia	26 (14.2%)	17 (15.3%)	9 (12.5%)	0.67
WIC	0.8 ± 1.1	0.7 ± 1.1	0.9 ± 1.0	0.17
Pre-stroke mRS	0 (0–1)	0 (0–1)	0 (0–1)	0.53
Initial severity, NIHSS	0 (0–2)	0 (0–2)	0 (0–2)	0.53
Baseline severity, NIHSS at 7 days	0 (0–1)	0 (0–1)	0 (0–1)	0.43
Duration of hospitalization, days	11.9 ± 14.0	12.6 ± 15.4	10.7 ± 11.6	0.35
Inpatient rehabilitation, yes	82 (44.8%)	52 (46.8%)	30 (41.7%)	0.54
Complications during hospitalization				
Thromboembolic disease, yes	1 (0.5%)	1 (0.9%)	0 (0%)	1
Pneumonia, yes	1 (0.5%)	1 (0.9%)	0(0%)	1
Urinary tract infection, yes	5 (2.7%)	2 (1.8%)	3 (4.2%)	0.38
Fall and injuries, yes	1 (0.5%)	1 (0.9%)	0(0%)	1
Recurrence of stroke	6 (3.3%)	3 (2.7%)	3 (4.2%)	0.68
Initial treatment				0.81
Conservative medical treatment	173 (94.5%)	104 (93.7%)	69 (95.8%)	
Thrombolysis or thrombectomy	6 (3.3%)	4 (3.6%)	2 (2.8%)	
Decompressive craniectomy	4 (2.2%)	3 (2.7%)	1 (1.4%)	

WIC, Charlson’s weighted comorbidity index; mRS, modified Rankin Scale; NIHSS, National Institutes of Health Stroke Scale; *p < 0.05, **p < 0.001, compared between young and old patients using independent t-test and chi-square test for numerical and categorical variables, respectively.

In the subgroup analysis categorized by age, the young patients had a significantly higher rate of current smoking (45.0%) and alcohol consumption (60.4%) compared to that of the old patients (smoking 12.5%; alcohol 41.7%). Vascular territories, infarct etiologies, gender, comorbidities, initial severity, stroke recurrence and initial treatments did not show significant differences between the young and old patients.

### Multifaceted functional outcomes from 7 days to 36 months after stroke

**Figure 2:**
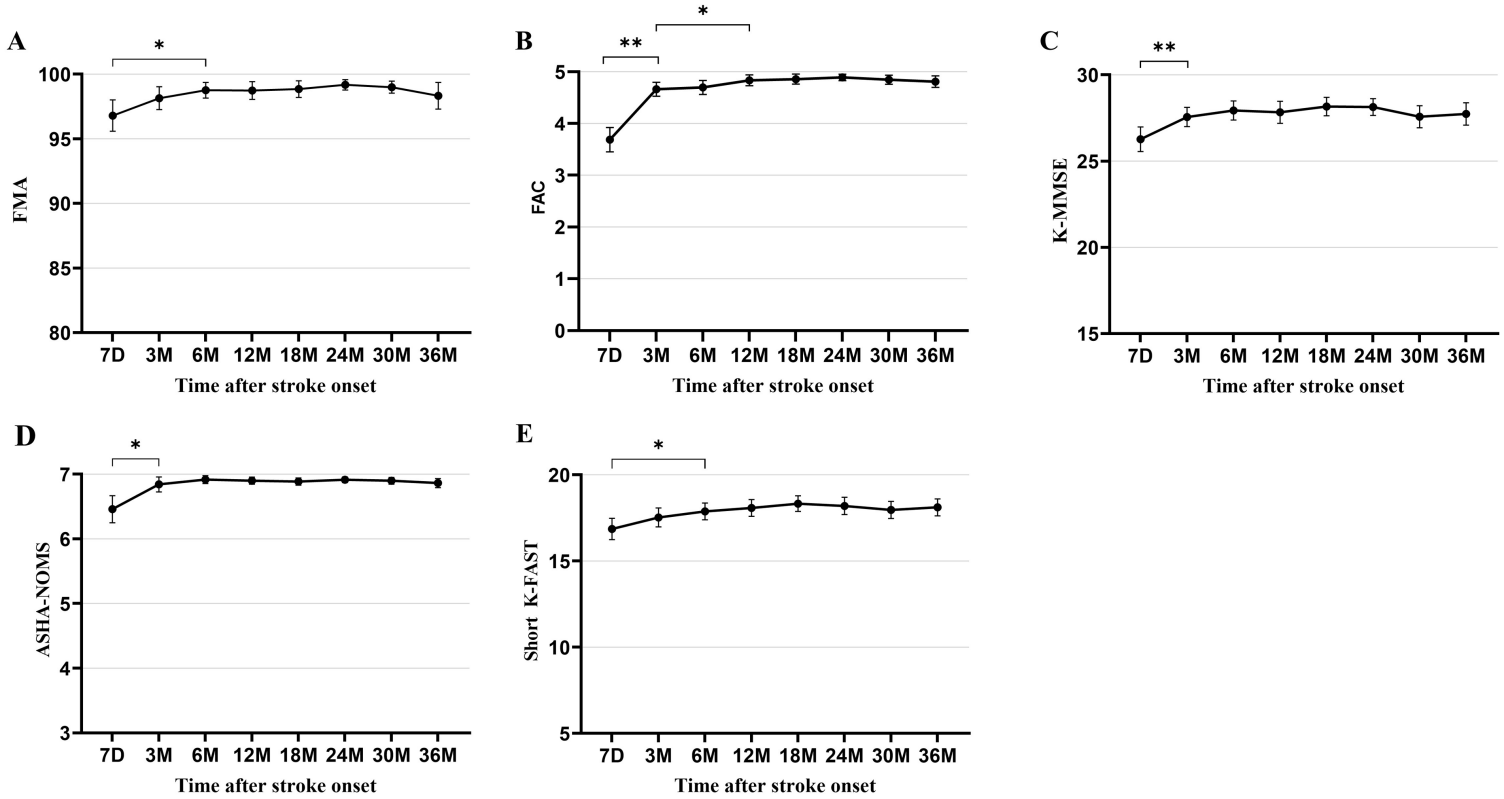
Multifaceted functional outcomes at each time point after stroke onset. **(A)** Fugl-Meyer Assessment. **(B)** Functional Ambulation Category. **(C)** Korean Mini-Mental State Examination. **(D)** American Speech-Language-Hearing Association National Outcome Measurement System Swallowing Scale. **(E)** Short version of the Korean Frenchay Aphasia Screening Test. All data are presented as mean values with 95% confidence intervals. *p < 0.05, **p < 0.001, compared between each time point using paired t-test and Wilcoxon signed-rank test, with Bonferroni correction.

All functional outcomes documented in patients with isolated cerebellar infarction showed significant improvement from baseline at 7 days after stroke and plateaued between 3 to 12 months after stroke, with variation observed for each functional domain. Motor and speech functions improved significantly from 7 days (mean [SD] FMA 96.8 ± 8.3; short K-FAST 16.9 ± 4.2) to 6 months (FMA 98.8 ± 3.8; short K-FAST 17.9 ± 3.1) after stroke and plateaued. Cognitive and swallowing function improved significantly from 7 days (K-MMSE 26.3 ± 4.9; mean [SD] ASHA-NOMS 6.5 ± 1.4) to 3 months (K-MMSE 27.6 ± 3.6; ASHA-NOMS 6.8 ± 0.7) after stroke onset and plateaued. Ambulatory function, as measured by mean (SD) FAC score, improved significantly from 7 days (3.7 ± 1.6) to 3 months (4.7 ± 0.9). In addition, the mean (SD) FAC score showed further significant improvement at 12 months (4.8 ± 0.7) and then plateaued. This plateau of recovery in ambulatory function differed from that of other functional domains (Figure 2).

**Figure 3:**
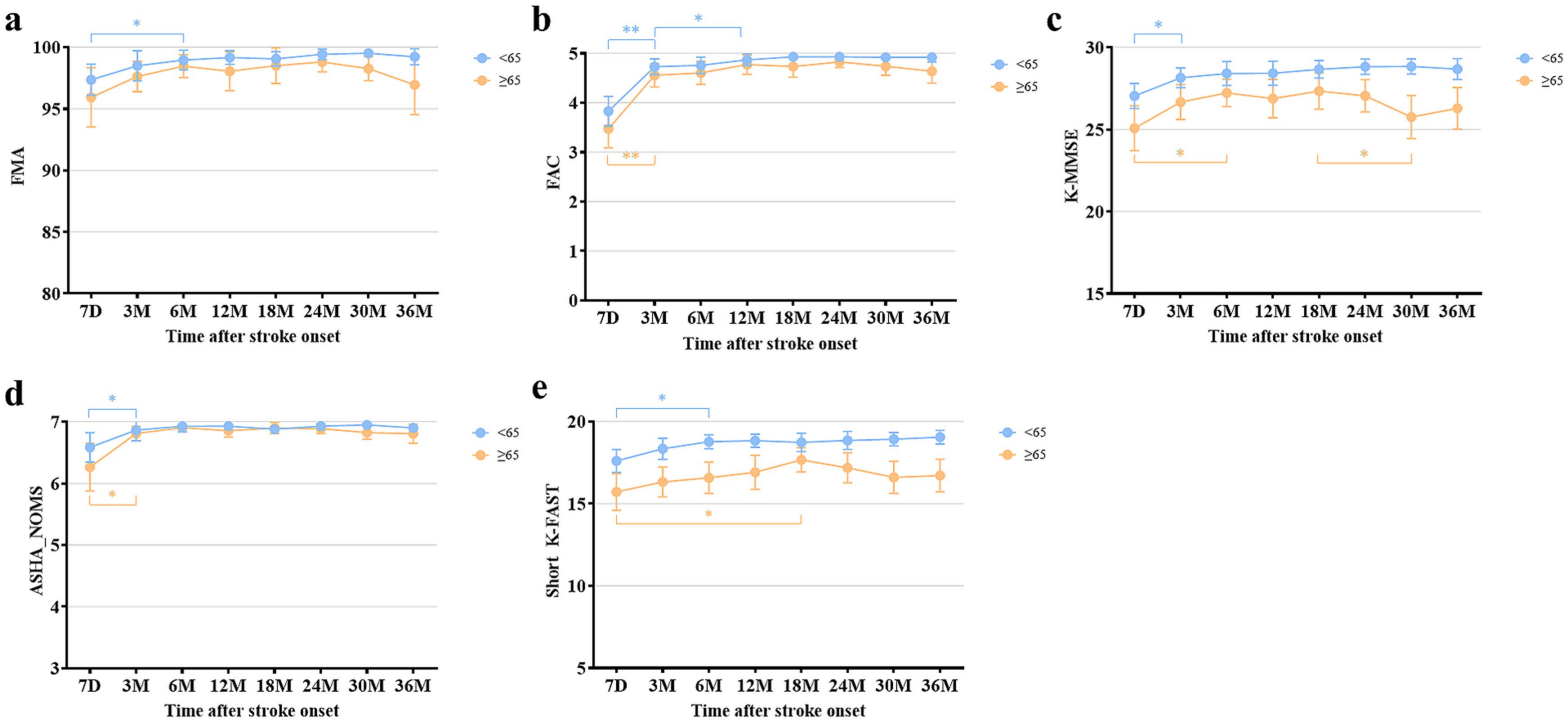
Comparison of multifaceted functional outcomes at each time point after stroke onset between the young and old patients. **(a)** Fugl-Meyer Assessment. **(b)** Functional Ambulation Category. **(c)** Korean Mini-Mental State Examination. **(d)** American Speech-Language-Hearing Association National Outcome Measurement System Swallowing Scale. **(e)** Short version of the Korean Frenchay Aphasia Screening Test. All data are presented as mean values with 95% confidence intervals. *p < 0.05, **p < 0.001, compared between each time point using paired t-test and Wilcoxon signed-rank test, with Bonferroni correction.

In subgroup analysis, all mean values of functional domains were better in the young patients than in the old patients. The mean scores of FMA and FAC showed significant differences between the two groups at 30 and 36 months after stroke. ASHA-NOMS showed significant differences at 30 months after onset. The mean scores of both K-MMSE and short K-FAST showed significant differences between the two groups at all follow-up time points (Supplementary Table S3). In the young patient group, all functional outcomes showed the plateau of recovery similar to that observed in the total patients, as analyzed by the paired t-tests (Figure 3). In the old patients, the plateau of recovery varied across the functional domains. The mean values of FMA showed no significant differences among all measures. FAC and ASHA-NOMS showed significant improvement from 7 days to 3 months and reached a plateau. In addition, a trend toward deterioration of mean values in motor, ambulation, and swallowing functions was observed from 24 months after stroke, although these findings did not reach statistical significance. Cognitive function improved significantly from 7 days to 6 months after stroke. Language function showed a recovery from 7 days to 18 months after stroke and a trend toward deterioration from 18 months onward, without statistical significance.

### Functional independence of patients with isolated cerebellar infarction

**Figure 4:**
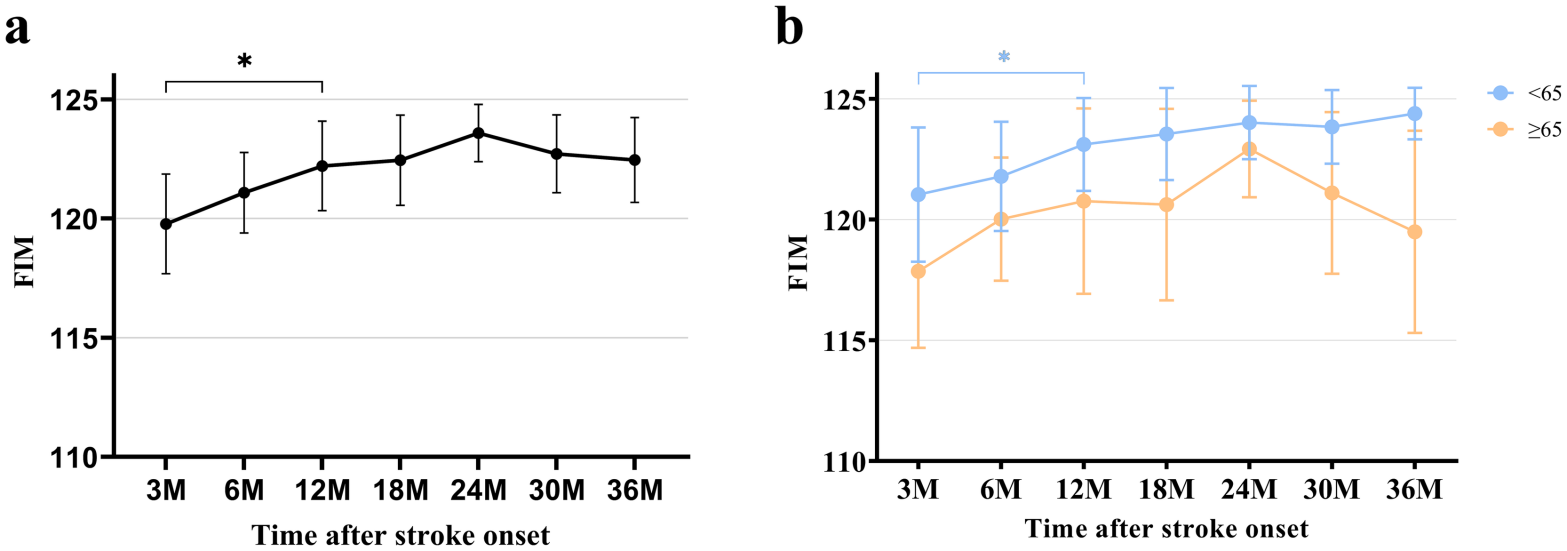
FIM at each time point after stroke. **(a)** FIM of total patients. **(b)** FIM categorized by age. All data are presented as mean values with 95% confidence intervals. FIM, Functional Independence Measure *p < 0.05, compared between each time point using paired t-test with Bonferroni correction.

In terms of functional independence, as measured by mean (SD) FIM score, there was a significant improvement from 3 months (119.8 ± 13.3) to 12 months (122.2 ± 12.0) after stroke and then reached a plateau, as analyzed by the paired t-test. In the young patients, mean FIM scores showed an increasing trend over time, although statistical significance was reached only between 3 and 12 months after stroke, as observed in total patients. In the old patients, a tendency toward deterioration in FIM was observed from 24 months after stroke without statistical significance. Furthermore, at 36 months after stroke onset, mean (SD) FIM scores showed significant difference between the young (124.4 ± 5.7) and old (119.5 ± 17.8) patients (Figure 4; Supplementary Table S3).

In multivariable linear regression analysis, using significant factors identified from the univariable analyses, vascular territory involving SCA, older age, female sex, and greater initial severity measured by NIHSS score were found to be negative prognostic factors predicting FIM at 12 months after stroke, when FIM reached a plateau (Table 2).

**Table 2:** Linear regression model predicting FIM at 12 months after stroke.

Factors	Univariable analysis			Multivariable analysis^†^		
	β	SE	*p-*value	β	SE	*p-*value
Vascular territory (reference, PICA)						
Superior cerebellar artery	−6.08	2.91	0.04^*^	−5.49	2.64	0.04^*^
Age	−0.21	0.08	0.01^*^	−0.17	0.08	0.03^*^
Sex, female	−7.31	2.11	<0.001^**^	−6.59	2.25	0.004^*^
Alcohol, current	4.57	1.88	0.02^*^	1.13	1.94	0.56
Initial severity, NIHSS	−1.81	0.41	<0.001^**^	−1.60	0.43	<0.001^**^
Duration of hospitalization, days	−0.15	0.06	0.02^*^	−0.05	0.07	0.52
Functional level at 7 days						
FMA	0.28	0.11	0.02^*^	−0.09	0.15	0.56
FAC	1.88	0.57	0.001^*^	0.19	0.78	0.81
K-MMSE	0.57	0.21	0.01^*^	0.03	0.24	0.88
ASHA-NOMS	1.81	0.65	0.01^*^	0.70	0.86	0.42

FIM, Functional Independence Measurement; SE, standard error, PICA, posterior inferior cerebellar artery; NIHSS, National Institutes of Health Stroke Scale; FMA, Fugl-Meyer Assessment; FAC, Functional Ambulation Category; K-MMSE, Korean Mini-Mental State Examination; ASHA-NOMS, American Speech-Language-Hearing Association National Outcome Measurement System Swallowing Scale. *p < 0.05, **p < 0.001.

† *R2 0.26 in multivariable linear regression analysis.*

## Discussion

This study demonstrated the long-term functional recovery across various functional domains in patients with isolated cerebellar infarction, showing that recovery plateaued between 3 and 12 months after onset. Additionally, functional independence showed significant improvement up to 12 months after onset and a relatively favorable prognosis up to 36 months.

The clinical presentation of cerebellar infarction has been well documented in previous studies (1, 2, 6). Regarding the plateau of recovery, previous studies have reported that the recovery of functional levels in stroke patients generally plateau between 6 and 18 months (29–32). In this study, patients with cerebellar infarction showed relatively faster rate of recovery. Cognitive and swallowing functions plateaued at 3 months, motor and language functions at 6 months, while ambulatory function significantly improved up to 12 months (Figure 2). Previous studies have also reported that the cognitive deficits and dysphagia recover relatively faster than other functional domains, which aligns with the findings this study (33, 34). Regarding motor function, previous studies have reported that upper extremity function recovered relatively faster, with one study indicating that the majority of gains occurred within the first 2 weeks after onset (9, 35). Other study by Huynh et al., reported that upper limb motor function continued to improved up to 1-year after onset and suggested that changes of cortical excitability persisted throughout the follow-up period (36). Additionally, another study suggested that lower limb ataxia improved up to 3 months, although incoordination of the lower limb remained (37). In this study, we demonstrated that motor function, as evaluated by the FMA, plateaued at 6 months after onset. This finding may be explained by the faster recovery of upper limb function, followed by the gradual improvement of lower limb function, including ataxia. Furthermore, this study also demonstrated continued improvement in ambulatory function up to 12 months. This improvement in gait may be beyond balance problems, including further improvement in gait speed, which was reported as the remaining deficit in the previous study (37). Future research is needed to identify the precise factors that contribute to the improvement in motor and ambulatory function. The recovery of language function in patients with cerebellar infarction is still not well recognized. Recently, language deficits resulting from cerebellar lesions have been associated with cognitive dysfunction or non-motor language impairments (38–40). Such deficits include difficulties in word retrieval, impairments in reading and writing, reduced fluency, or agrammatic speech. However, the exact longitudinal trajectory of cerebellar-induced language deficits remains unclear. This study demonstrated that language deficits in patients with cerebellar infarction recovered within 6 months after onset. However, the details of recovery in these deficits require further investigation in future studies.

In subgroup analysis, the young patients had better outcomes as indicated by the mean values across all functional domains. Notably, significant differences were observed in cognitive functions. Cognitive function significantly declined between 18 and 30 months after onset, as determined by the paired t-test. One study reported a long-term cognitive deficit in 277 young ischemic stroke patients, including 24 patients involving cerebellum (41). They reported impairments in mental speed, cognitive flexibility, and working memory. Other study reported that cerebellar infarction patients may exhibit disturbances of executive function and verbal fluency (42). These cognitive defects have been reported in previous studies as cerebellar cognitive affective syndrome (CCAS) (43, 44). On the other hand, while not yet definitively established, prior studies have reported on vascular cognitive impairment (VCI) following cerebellar stroke (45, 46). In our study, we observed a trend of cognitive decline in old patients, potentially indicating increased susceptibility to CCAS or VCI. Further research is needed to clarify the mechanisms behind long-term cognitive dysfunction in cerebellar infarction patients. Language function, as measured by the short K-FAST also showed a deteriorating trend in the old patients, although statistical significance was not found. Since the short K-FAST is primarily developed to assess aphasia, it may not sufficiently capture the broader spectrum of language dysfunctions observed in patients with cerebellar infarction, such as agrammatism, dysarthria, or deficits secondary to cognitive impairment (39, 47, 48). Nevertheless, this deteriorating trend in the old patients may suggest that the long-term language problems induced by cerebellar infarction warrant further investigation using more detailed assessments and exploring their relationship with cognitive function. Swallowing dysfunction, or dysphagia, showed no significant differences in recovery between the two subgroups, both of which exhibited favorable outcomes. Since dysphagia is relatively less frequently reported as a disabling problem in patients with cerebellar lesions, the finding of this study align with that of previous research (49). Regarding motor function, as assessed by FMA, the old patients did no show significant differences throughout the follow-up period, although a declining trend was observed starting at 24 months post-onset without statistical significance. The young patients reached a plateau at 6 months and maintained a favorable status. The deteriorating trend observed in the old patients may warrant further investigation in future studies, including a detailed assessment of cerebellar-specific motor symptoms, such as limb ataxia.

Functional independence, as measured by FIM, significantly improved from 3 to 12 months after onset, possibly due to improvements in ambulatory function. This result may suggest that the level of independence in patients with cerebellar infarction is primarily influenced by ambulatory function, and consistent rehabilitation efforts should focus on improving ambulatory function. In subgroup analysis, young patients showed improvement from 3 to 12 months after onset. Moreover, FIM demonstrated a sustained improving tendency up to 36 months without statistical significance. However, the mean value of FIM score in old patients exhibited no significant differences during the follow-up period. In addition, a declining trend in independence from 24 months was observed, although this trend did not reach statistical significance. This may be associated with deteriorations in the domains of motor, ambulation, and cognition. A previous study reported that the functional status of stroke patients, as measured by the Barthel Index, showed a decline beginning 3 years after onset. This decline was associated with older age, and cognitive dysfunction was also suggested to contribute to functional decline (50). Further investigation and research are required to evaluate these relationships in detail.

Furthermore, we demonstrated prognostic factors that could be associated with the functional outcome at 12 months, when the plateau was reached. Older age, vascular territory involving SCA, female sex, and greater initial severity were found to be negative prognostic factors. Well-known prognostic factors documented in previous studies included the initial severity, loss of consciousness, stroke volume, and the vascular territories (2, 6, 10, 13, 51). Consistent with previous studies, patients with lesions in the SCA vascular territories showed worse prognosis, which is well-documented due to more severe impairments in posture and ataxia (2, 10, 37). In addition, we revealed that age is an important prognostic factor for cerebellar infarction. Although older age is a well-known negative prognostic factor in stroke, the impact of age on cerebellar infarction has not been well established. One previous study reported that age had no significant effect on functional recovery from cerebellar infarction (6). On the other hand, a recent study reported old age as an independent predictor for unfavorable outcomes (13). We suggest that age should be focused when addressing the functional outcome in cerebellar infarction. Furthermore, sex was identified as a prognostic factor. Prior research indicates that female patients with general stroke tend to have worse functional outcomes than males, however, the exact underlying mechanisms for this disparity have not been fully understood (52, 53). Future studies would focus on exploring this relationship in cerebellar infarction patients.

The strength of this study is that it was a relatively large, multicenter study evaluating the long-term functional outcomes of various functional domains. In addition, we examined the functional outcomes in more detail and showed the differences based on age. This study has several limitations. First, radiologic information was lacking. However, this study aimed to demonstrate the recovery based on multifaceted functional outcomes. Thus, we believe the results are clinically meaningful despite the absence of radiologic findings. Second, because these data were collected from the KOSCO study, which assessed stroke patients in general, cortical stroke-centric tools were used in the assessments. As a result, cerebellar-specific issues such as balance problems, dysmetria, or cerebellar-specific dysarthria may not have been adequately captured. Additionally, the tools used in this study are thought to have ceiling effects, potentially leading to an underestimation of actual recovery. Therefore, future research is needed to emphasize the importance of incorporating cerebellar-specific assessments to address this limitation. Third, there were relatively few patients received thrombolysis or thrombectomy. Therefore, the impact of these reperfusion therapies on the long-term functional outcome remains unknown. Future studies are needed to evaluate the impact in a larger population, although recent study demonstrated receiving reperfusion therapies was not an independent predictor of the outcome (13). Fourth, due to low predictive power and the relatively small number of patients reporting functional dependency, the results maybe imprecise in terms of clinical applicability in predicting functional prognosis (54). Further studies involving a larger number of participants with more parameters at subacute stroke phase will be needed to accurately evaluate the impact of functional prognosis. Lastly, there were missing data at each time point during the follow-up period.

## Conclusion

In conclusion, this study demonstrated the recovery of various functional domains in patients with isolated cerebellar infarction. The long-term prognosis of patients with cerebellar infarction was relatively favorable. Furthermore, old patients with isolated cerebellar infarction tended to have a poor prognosis and showed deteriorating trends in their functional outcomes from 18 to 24 months after stroke. This study provided valuable information for the evaluation and management of patients with isolated cerebellar infarction.

## Data Availability

The original contributions presented in the study are included in the article/Supplementary material, further inquiries can be directed to the corresponding authors.

## References

[1] Macdonell RA Kalnins RM Donnan GA. Cerebellar infarction: natural history, prognosis, and pathology. Stroke. (1987) 18:849–55. doi: 10.1161/01.STR.18.5.849, PMID: 3629642

[2] Tohgi H Takahashi S Chiba K Hirata Y. Cerebellar infarction. Clinical and neuroimaging analysis in 293 patients. The Tohoku cerebellar infarction study group. Stroke. (1993) 24:1697–701. doi: 10.1161/01.STR.24.11.1697, PMID: 8236346

[3] Moulin T Tatu L Vuillier F Berger E Chavot D Rumbach L. Role of a stroke data bank in evaluating cerebral infarction subtypes: patterns and outcome of 1,776 consecutive patients from the Besancon stroke registry. Cerebrovasc Dis. (2000) 10:261–71. doi: 10.1159/000016068, PMID: 10878430

[4] Savitz SI Caplan LR Edlow JA. Pitfalls in the diagnosis of cerebellar infarction. Acad Emerg Med. (2007) 14:63–8. doi: 10.1197/j.aem.2006.06.060, PMID: 17200515

[5] Edlow JA Newman-Toker DE Savitz SI. Diagnosis and initial management of cerebellar infarction. Lancet Neurol. (2008) 7:951–64. doi: 10.1016/S1474-4422(08)70216-3, PMID: 18848314

[6] Wright J Huang C Strbian D Sundararajan S. Diagnosis and management of acute cerebellar infarction. Stroke. (2014) 45:e56–8. doi: 10.1161/STROKEAHA.114.004474, PMID: 39931041

[7] Jauss M Krieger D Hornig C Schramm J Busse O Centers GS. Surgical and medical management of patients with massive cerebellar infarctions: results of the German-Austrian Cerebellar infarction study. J Neurol. (1999) 246:257–64. doi: 10.1007/s004150050344, PMID: 10367693

[8] Kelly P Stein J Shafqat S Eskey C Doherty D Chang Y. Functional recovery after rehabilitation for cerebellar stroke. Stroke. (2001) 32:530–4. doi: 10.1161/01.STR.32.2.530, PMID: 11157193

[9] Picelli A Zuccher P Tomelleri G Bovi P Moretto G Waldner A. Prognostic importance of lesion location on functional outcome in patients with cerebellar ischemic stroke: a prospective pilot study. Cerebellum. (2017) 16:257–61. doi: 10.1007/s12311-015-0757-6, PMID: 26758032

[10] Nickel A Cheng B Pinnschmidt H Arpa E Ganos C Gerloff C. Clinical outcome of isolated cerebellar stroke—a prospective observational study. Front Neurol. (2018) 9:580. doi: 10.3389/fneur.2018.00580, PMID: 30065696 PMC6056646

[11] Grips E Sedlaczek O Hr B Fritzinger M Daffertshofer M Hennerici M. Supratentorial age-related white matter changes predict outcome in cerebellar stroke. Stroke. (2005) 36:1988–93. doi: 10.1161/01.STR.0000177869.02361.dc, PMID: 16081861

[12] Liew S-L Schweighofer N Cole JH Zavaliangos-Petropulu A Tavenner BP Han LK. Association of brain age, lesion volume, and functional outcome in patients with stroke. Neurology. (2023) 100:e2103–13. doi: 10.1212/WNL.0000000000207219, PMID: 37015818 PMC10186236

[13] Won S-Y Melkonian R Behmanesh B Bernstock JD Czabanka M Dubinski D. Cerebellar stroke score and grading scale for the prediction of mortality and outcomes in ischemic Cerebellar stroke. Stroke. (2023) 54:2569–75. doi: 10.1161/STROKEAHA.123.043478, PMID: 37551591

[14] Chang WH Sohn MK Lee J Kim DY Lee S-G Shin Y-I. Korean stroke cohort for functioning and rehabilitation (KOSCO): study rationale and protocol of a multi-Centre prospective cohort study. BMC Neurol. (2015) 15:1–7. doi: 10.1186/s12883-015-0293-5, PMID: 25886039 PMC4376073

[15] Adams HP Jr Bendixen BH Kappelle LJ Biller J Love BB Gordon DL. Classification of subtype of acute ischemic stroke. Definitions for use in a multicenter clinical trial. TOAST. trial of org 10172 in acute stroke treatment. Stroke. (1993) 24:35–41. doi: 10.1161/01.STR.24.1.35, PMID: 7678184

[16] Kleindorfer DO Towfighi A Chaturvedi S Cockroft KM Gutierrez J Lombardi-Hill D. 2021 guideline for the prevention of stroke in patients with stroke and transient ischemic attack: a guideline from the American Heart Association/American Stroke Association. Stroke. (2021) 52:e364–467. doi: 10.1161/STR.0000000000000375, PMID: 34024117

[17] Tessier A Finch L Daskalopoulou SS Mayo NE. Validation of the Charlson comorbidity index for predicting functional outcome of stroke. Arch Phys Med Rehabil. (2008) 89:1276–83. doi: 10.1016/j.apmr.2007.11.049, PMID: 18586129

[18] Burn J. Reliability of the modified Rankin scale. Stroke. (1992) 23:438. doi: 10.1161/str.23.3.438b 1610453

[19] Oh MS Yu K-H Lee J-H Jung S Ko I-S Shin J-H. Validity and reliability of a Korean version of the national institutes of health stroke scale. J Clin Neurol. (2012) 8:177–83. doi: 10.3988/jcn.2012.8.3.177, PMID: 23091526 PMC3469797

[20] Sanford J Moreland J Swanson LR Stratford PW Gowland C. Reliability of the Fugl-Meyer assessment for testing motor performance in patients following stroke. Phys Ther. (1993) 73:447–54. doi: 10.1093/ptj/73.7.447, PMID: 8316578

[21] Mehrholz J Wagner K Rutte K Meiβner D Pohl M. Predictive validity and responsiveness of the functional ambulation category in hemiparetic patients after stroke. Arch Phys Med Rehabil. (2007) 88:1314–9. doi: 10.1016/j.apmr.2007.06.764, PMID: 17908575

[22] Kang Y Na D-L Hahn S. A validity study on the Korean Mini-mental state examination (K-MMSE) in dementia patients. J Korean Neurol Assoc. (1997) 15:300–8.

[23] Wesling M Brady S Jensen M Nickell M Statkus D Escobar N. Dysphagia outcomes in patients with brain tumors undergoing inpatient rehabilitation. Dysphagia. (2003) 18:203–10. doi: 10.1007/s00455-002-0098-8, PMID: 14506986

[24] Pyun SB Hwang YM Ha JW Yi H Park KW Nam K. Standardization of Korean version of Frenchay aphasia screening test in normal adults. J Korean Acad Rehabilt Med. (2009) 33:436–40.

[25] Dodds TA Martin DP Stolov WC Deyo RA. A validation of the functional independence measurement and its performance among rehabilitation inpatients. Arch Phys Med Rehabil. (1993) 74:531–6. doi: 10.1016/0003-9993(93)90119-U, PMID: 8489365

[26] Rayegani SM Raeissadat SA Alikhani E Bayat M Bahrami MH Karimzadeh A. Evaluation of complete functional status of patients with stroke by functional Independence measure scale on admission, discharge, and six months poststroke. Iran J Neurol. (2016) 15:202–8. PMID: 28435628 PMC5392193

[27] Kunkel SR Brown JS Whittington FJ. Global aging: Comparative perspectives on aging and the life course. New York: Springer Publishing Company (2014).

[28] Yousufuddin M Young N. Aging and ischemic stroke. Aging (Albany NY). (2019) 11:2542–4. doi: 10.18632/aging.101931, PMID: 31043575 PMC6535078

[29] Langhorne P Coupar F Pollock A. Motor recovery after stroke: a systematic review. Lancet Neurol. (2009) 8:741–54. doi: 10.1016/S1474-4422(09)70150-4, PMID: 19608100

[30] Langhorne P Bernhardt J Kwakkel G. Stroke rehabilitation. Lancet. (2011) 377:1693–702. doi: 10.1016/S0140-6736(11)60325-5, PMID: 21571152

[31] Shin S Lee Y Chang WH Sohn MK Lee J Kim DY. Multifaceted assessment of functional outcomes in survivors of first-time stroke. JAMA Netw Open. (2022) 5:3094. doi: 10.1001/jamanetworkopen.2022.33094, PMID: 36149652 PMC9508656

[32] Lee HS Sohn MK Lee J Kim DY Shin YI Oh GJ. Long-term functional outcome in patients with isolated thalamic stroke: the KOSCO study. J Am Heart Assoc. (2024) 13:e032377. doi: 10.1161/JAHA.123.032377, PMID: 38348806 PMC11010118

[33] Suzuki M Sugimura Y Yamada S Omori Y Miyamoto M Yamamoto J-i. Predicting recovery of cognitive function soon after stroke: differential modeling of logarithmic and linear regression. PLoS One. (2013) 8:e53488. doi: 10.1371/journal.pone.0053488, PMID: 23326439 PMC3543398

[34] González-Fernández M Ottenstein L Atanelov L Christian AB. Dysphagia after stroke: an overview. Curr Phys Med Rehabil Rep. (2013) 1:187–96. doi: 10.1007/s40141-013-0017-y, PMID: 24977109 PMC4066736

[35] Jr K Pierscianek D Hirsiger S Bultmann U Schoch B Gizewski ER. Recovery of upper limb function after cerebellar stroke: lesion symptom mapping and arm kinematics. Stroke. (2010) 41:2191–200. doi: 10.1161/STROKEAHA.110.583641, PMID: 20814010

[36] Huynh W Krishnan AV Vucic S Lin CS Kiernan MC. Motor cortex excitability in acute cerebellar infarct. Cerebellum. (2013) 12:826–34. doi: 10.1007/s12311-013-0493-8, PMID: 23728898

[37] Bultmann U Pierscianek D Gizewski ER Schoch B Fritsche N Timmann D. Functional recovery and rehabilitation of postural impairment and gait ataxia in patients with acute cerebellar stroke. Gait Posture. (2014) 39:563–9. doi: 10.1016/j.gaitpost.2013.09.011, PMID: 24119775

[38] Murdoch BE. The cerebellum and language: historical perspective and review. Cortex. (2010) 46:858–68. doi: 10.1016/j.cortex.2009.07.018, PMID: 19828143

[39] Satoer D Koudstaal PJ Visch-Brink E van der Giessen RS. Cerebellar-induced aphasia after stroke: evidence for the “linguistic cerebellum”. Cerebellum. (2024) 23:1457–65. doi: 10.1007/s12311-024-01658-1, PMID: 38244134 PMC11269354

[40] Li H Xie X. Cerebellar activity and functional connectivity in subacute subcortical aphasia: association with language recovery. Neuroscience. (2025) 565:320–6. doi: 10.1016/j.neuroscience.2024.11.077, PMID: 39626825

[41] Schaapsmeerders P Maaijwee NA van Dijk EJ Rutten-Jacobs LC Arntz RM Schoonderwaldt HC. Long-term cognitive impairment after first-ever ischemic stroke in young adults. Stroke. (2013) 44:1621–8. doi: 10.1161/STROKEAHA.111.000792, PMID: 23652272

[42] Liu Q Liu C Chen Y Zhang Y. Cognitive dysfunction following cerebellar stroke: insights gained from neuropsychological and neuroimaging research. Neural Plast. (2022) 2022:1–11. doi: 10.1155/2022/3148739, PMID: 35465397 PMC9033331

[43] Schmahmann JD. Vascular syndromes of the thalamus. Stroke. (2003) 34:2264–78. doi: 10.1161/01.STR.0000087786.38997.9E, PMID: 12933968

[44] Ahmadian N van Baarsen K van Zandvoort M Robe PA. The cerebellar cognitive affective syndrome—a meta-analysis. Cerebellum. (2019) 18:941–50. doi: 10.1007/s12311-019-01060-2, PMID: 31392563 PMC6761084

[45] Sui R Zhang L. Cerebellar dysfunction may play an important role in vascular dementia. Med Hypotheses. (2012) 78:162–5. doi: 10.1016/j.mehy.2011.10.017, PMID: 22075237

[46] Mijajlović MD Pavlović A Brainin M Heiss W-D Quinn TJ Ihle-Hansen HB. Post-stroke dementia–a comprehensive review. BMC Med. (2017) 15:1–12. doi: 10.1186/s12916-017-0779-7, PMID: 28095900 PMC5241961

[47] Hoche F Guell X Vangel MG Sherman JC Schmahmann JD. The cerebellar cognitive affective/Schmahmann syndrome scale. Brain. (2018) 141:248–70. doi: 10.1093/brain/awx317, PMID: 29206893 PMC5837248

[48] Vlasova RM Panikratova YR Pechenkova EV. Systematic review and meta-analysis of language symptoms due to cerebellar injury. Cerebellum. (2023) 22:1274–86. doi: 10.1007/s12311-022-01482-5, PMID: 36205825

[49] Rönnefarth M Hanisch N Brandt AU Mähler A Endres M Paul F. Dysphagia affecting quality of life in cerebellar ataxia—a large survey. Cerebellum. (2020) 19:437–45. doi: 10.1007/s12311-020-01122-w, PMID: 32170655 PMC7198478

[50] Dhamoon MS Moon YP Paik MC Boden-Albala B Rundek T Sacco RL. Long-term functional recovery after first ischemic stroke: the northern Manhattan study. Stroke. (2009) 40:2805–11. doi: 10.1161/STROKEAHA.109.549576, PMID: 19556535 PMC2830874

[51] Cano L Cardona P Quesada H Mora P Rubio F. Cerebellar infarction: prognosis and complications of vascular territories. Neurología. (2012) 27:330–5. doi: 10.1016/j.nrleng.2012.07.010 22341984

[52] Bonkhoff AK Schirmer MD Bretzner M Hong S Regenhardt RW Brudfors M. Outcome after acute ischemic stroke is linked to sex-specific lesion patterns. Nat Commun. (2021) 12:3289. doi: 10.1038/s41467-021-23492-3, PMID: 34078897 PMC8172535

[53] Ryu W-S Chung J Schellingerhout D Jeong S-W Kim H-R Park JE. Biological mechanism of sex difference in stroke manifestation and outcomes. Neurology. (2023) 100:e2490–503. doi: 10.1212/WNL.0000000000207346, PMID: 37094993 PMC10264052

[54] Hamilton D Ghert M Simpson A. Interpreting regression models in clinical outcome studies. Bone Joint Res. (2015) 4:152–3. doi: 10.1302/2046-3758.49.2000571, PMID: 26392591 PMC4678365

