# Supplementary material for: Long-term functional outcomes in patients with isolated cerebellar infarction: the KOSCO study
Source: Front Neurol. 2025 Mar 10;16:1541245. doi: 10.3389/fneur.2025.1541245 (PMC11930828; doi:10.3389/fneur.2025.1541245)
Supplement: Supplementary file 1 [file Supplementary_file_1.docx]

**Supplementary materials**

**Number of tables: 3**

**Table S1.** IRB approval numbers of participating hospitals.

| **Institute** | **IRB approval numbers** |
| --- | --- |
| Samsung Medical Center | 2012-06-016 |
| Severance Hospital | 4-2012-0341 |
| Konkuk University Medical Center | 1180-01-700 |
| Chungnam National University Hospital | 2012-06-011 |
| Chonnam National University Hospital | CNUH-2012-127 |
| Pusan National University Yangsan Hospital | 05-2012-057 |
| Kyungpook National University Hospital | 2013-03-029 |
| Wonkwang University Hospital | WKUH-1515 |
| Jeju National University Hospital | 2013-02-001 |

IRB, Institutional review board.

**Table S2.** Demographic and clinical characteristics of the included and excluded patients.

| **Demographic and clinical characteristics** | **No.(%) or mean±SD or median (IQR)** | | | | ***p*-value** |
| --- | --- | --- | --- | --- | --- |
|  | **Total (n=277)** | **Included (n=183)** | **Lost**  **(n=77)** | **Death (n=17)** |  |
| Lesion side |  |  |  |  | 0.06 |
| Right | 114 (41.2%) | 71 (38.8%) | 37 (48.1%) | 6 (35.3%) |  |
| Left | 127 (45.8%) | 91 (49.7%) | 31 (40.3%) | 5 (29.4%) |  |
| Bilateral | 36 (13.0%) | 21 (11.5) | 9 (11.7%) | 6 (35.3%) |  |
| Vascular territory |  |  |  |  | 0.08 |
| Superior cerebellar artery | 28 (10.1%) | 22 (12.0%) | 3 ( 3.9%) | 3 (17.6%) |  |
| Anterior inferior cerebellar artery | 16 (5.8%) | 7 (3.8%) | 8 (10.4%) | 1 ( 5.9%) |  |
| Posterior inferior cerebellar artery | 225 (81.2%) | 147 (80.3%) | 65 (84.4%) | 13 (76.5%) |  |
| Multiple involvement | 8 (2.9%) | 7 (3.8%) | 1 ( 1.3%) | 0 ( 0.0%) |  |
| Etiology |  |  |  |  | 0.80 |
| Large artery artherosclerosis | 101 (36.5%) | 70 (38.3%) | 25 (32.5%) | 6 (35.3%) |  |
| Small vessel occlusion | 73 (26.4%) | 46 (25.1%) | 23 (29.9%) | 4 (23.5%) |  |
| Cardioembolism | 42 (15.2%) | 25 (13.7%) | 12 (15.6%) | 5 (29.4%) |  |
| Other determined | 27 (9.7%) | 20 (10.9%) | 6 ( 7.8%) | 1 ( 5.9%) |  |
| Undetermined | 34 (12.3%) | 22 (12.0%) | 11 (14.3%) | 1 ( 5.9%) |  |
| Age | 62.2±13.2 | 61.0±11.8 | 61.9±15.1 | 76.9±9.6 | <0.001^**^ |
| Sex, male | 199 (71.8%) | 138 (75.4%) | 49 (63.6%) | 12 (70.6%) | 0.15 |
| Body mass index | 23.8±3.1 | 24.0±3.1 | 23.7±2.9 | 21.3±2.8 | 0.004^*^ |
| Smoking, current | 82 (29.6%) | 59 (32.2%) | 22 (28.6%) | 1 ( 5.9%) | 0.07 |
| Alcohol, current | 132 (47.7%) | 97 ( 53.0%) | 30 (39.0%) | 5 (29.4%) | 0.04 |
| Risk factors |  |  |  |  |  |
| HTN | 155 (56.0%) | 103 (56.3%) | 39(50.6%) | 13 (76.5%) | 0.15 |
| DM | 65 (23.5%) | 40 (21.9%) | 20 (26.0%) | 5 (29.4%) | 0.60 |
| Coronary heart disease | 22 (7.9%) | 13 ( 7.1%) | 6 (7.8%) | 3 (17.6%) | 0.24 |
| Atrial fibrillation | 29 (10.5%) | 16 ( 8.7%) | 6 (7.8%) | 7 (41.2%) | 0.001^*^ |
| Hyperlipidemia, | 40 (14.4%) | 26 (14.2%) | 13(16.9%) | 1 (5.9%) | 0.57 |
| WIC | 0.8±1.2 | 0.8±1.1 | 0.6±0.8 | 1.7±3.0 | 0.01^*^ |
| Pre-stroke mRS | 0 (0-1) | 0 (0-1) | 0 (0-1) | 1 (0-3) | 0.01^*^ |
| Initial severity, NIHSS | 0 (0-2) | 0 (0-2) | 0 (0-2) | 2 (0-3) | 0.26 |
| Baseline severity, NIHSS at 7 days | 0 (0-1) | 0 (0-1) | 0 (0-1) | 2 (0-7) | 0.004^*^ |
| Duration of hospitalization, days | 12.3±13.9 | 11.9±14.0 | 11.7±11.8 | 20.1±19.1 | 0.002^*^ |
| Inpatient rehabilitation, yes | 126 (45.5%) | 82 (44.8%) | 31 (40.3%) | 13 (76.5%) | 0.02^*^ |
| Complications during hospitalization |  |  |  |  |  |
| Thromboembolic disease, yes | 1 (0.4%) | 1 ( 0.5%) | 0 (0%) | 0 (0%) | 1 |
| Pneumonia, yes | 5 (1.8%) | 1 ( 0.5%) | 1( 1.3%) | 3 (17.6%) | 0.001^*^ |
| Urinary tract infection, yes | 6 (2.2%) | 5 ( 2.7%) | 0 (0%) | 1 ( 5.9%) | 0.18 |
| Fall and injuries, yes | 1 (0.4%) | 1 ( 0.5%) | 0 (0%) | 0 (0%) | 1 |

WIC, Charlson’s weighted comorbidity index; mRS, modified Rankin Scale; NIHSS, National Institutes of Health Stroke Scale;

**p*<0.05, ***p*<0.001, compared between included patients, lost to follow-up patients and died during the follow-up period using Kruskal-Wallis test.

**Table S3.** Multifaceted functional outcomes at each time point after stroke.

| **Variable** | **Total** | | | **Young (<65)** | | **Old (≥65)** | | ***p*-value** |
| --- | --- | --- | --- | --- | --- | --- | --- | --- |
|  | **mean ±SD** | | **N** | **mean ±SD** | **N** | **mean ±SD** | **N** |  |
| FMA at 7D | | 96.8±8.3 | 183 | 97.4±6.7 | 111 | 95.9±10.3 | 72 | 0.26 |
| FMA at 3M | | 98.1±5.6 | 159 | 98.5±6.0 | 96 | 97.6±4.9 | 63 | 0.34 |
| FMA at 6M | | 98.8±3.8 | 158 | 99.0±3.9 | 95 | 98.5±3.7 | 63 | 0.43 |
| FMA at 12M | | 98.7±4.4 | 161 | 99.2±2.8 | 99 | 98.0±6.2 | 62 | 0.12 |
| FMA at 18M | | 98.8±4.1 | 160 | 99.0±3.0 | 100 | 98.5±5.6 | 60 | 0.42 |
| FMA at 24M | | 99.2±2.6 | 163 | 99.4±2.2 | 100 | 98.8±3.2 | 63 | 0.14 |
| FMA at 30M | | 99.0±3.0 | 168 | 99.5±1.9 | 99 | 98.3±4.1 | 69 | 0.008^*^ |
| FMA at 36M | | 98.3±7.1 | 183 | 99.2±3.5 | 111 | 96.9±10.3 | 72 | 0.03^*^ |
| FAC at 7D | | 3.7±1.6 | 183 | 3.8±1.6 | 111 | 3.5±1.6 | 72 | 0.14 |
| FAC at 3M | | 4.7±0.9 | 159 | 4.7±0.8 | 96 | 4.6±0.9 | 63 | 0.21 |
| FAC at 6M | | 4.7±0.9 | 158 | 4.8±0.8 | 95 | 4.6±0.9 | 63 | 0.27 |
| FAC at 12M | | 4.8±0.7 | 161 | 4.9±0.6 | 99 | 4.8±0.8 | 62 | 0.39 |
| FAC at 18M | | 4.9±0.6 | 160 | 4.9±0.4 | 100 | 4.7±0.8 | 60 | 0.05 |
| FAC at 24M | | 4.9±0.4 | 163 | 4.9±0.4 | 100 | 4.8±0.5 | 63 | 0.10 |
| FAC at 30M | | 4.8±0.6 | 168 | 4.9±0.4 | 99 | 4.7±0.8 | 69 | 0.04^*^ |
| FAC at 36M | | 4.8±0.8 | 183 | 4.9±0.5 | 111 | 4.6±1.0 | 72 | 0.02^*^ |
| K-MMSE at 7D | | 26.3±4.9 | 183 | 27.0±4.1 | 111 | 25.1±5.8 | 72 | 0.01^*^ |
| K-MMSE at 3M | | 27.6±3.6 | 159 | 28.1±3.0 | 96 | 26.7±4.2 | 63 | 0.01^*^ |
| K-MMSE at 6M | | 27.9±3.5 | 158 | 28.4±3.6 | 95 | 27.2±3.3 | 63 | 0.04^*^ |
| K-MMSE at 12M | | 27.8±4.1 | 161 | 28.4±3.6 | 99 | 26.9±4.6 | 62 | 0.02^*^ |
| K-MMSE at 18M | | 28.2±3.4 | 160 | 28.7±2.7 | 100 | 27.3±4.3 | 60 | 0.02^*^ |
| K-MMSE at 24M | | 28.1±3.2 | 163 | 28.8±2.3 | 100 | 27.0±3.9 | 63 | <0.001^**^ |
| K-MMSE at 30M | | 27.6±4.2 | 168 | 28.8±2.3 | 99 | 25.8±5.5 | 69 | <0.001^**^ |
| K-MMSE at 36M | | 27.7±4.4 | 183 | 28.7±3.4 | 111 | 26.3±5.4 | 72 | <0.001^**^ |
| ASHA-NOMS at 7D | | 6.5±1.4 | 183 | 6.6±1.3 | 111 | 6.3±1.7 | 72 | 0.14 |
| ASHA-NOMS at 3M | | 6.8±0.7 | 159 | 6.9±0.9 | 96 | 6.8±0.5 | 63 | 0.65 |
| ASHA-NOMS at 6M | | 6.9±0.4 | 158 | 6.9±0.4 | 95 | 6.9±0.3 | 63 | 0.74 |
| ASHA-NOMS at 12M | | 6.9±0.4 | 161 | 6.9±0.3 | 99 | 6.9±0.4 | 62 | 0.20 |
| ASHA-NOMS at 18M | | 6.9±0.4 | 160 | 6.9±0.4 | 100 | 6.9±0.4 | 60 | 0.73 |
| ASHA-NOMS at 24M | | 6.9±0.3 | 163 | 6.9±0.3 | 100 | 6.9±0.3 | 63 | 0.36 |
| ASHA-NOMS at 30M | | 6.9±0.3 | 168 | 6.9±0.2 | 99 | 6.8±0.5 | 69 | 0.02^*^ |
| ASHA-NOMS at 36M | | 6.9±0.5 | 183 | 6.9±0.3 | 111 | 6.8±0.7 | 72 | 0.19 |
| Short K-KFAST at 7D | | 16.9±4.2 | 179 | 17.6±3.6 | 109 | 15.7±4.7 | 70 | 0.003^*^ |
| Short K-KFAST at 3M | | 17.5±3.5 | 158 | 18.3±3.2 | 95 | 16.3±3.6 | 63 | <0.001^**^ |
| Short K-KFAST at 6M | | 17.9±3.1 | 156 | 18.8±2.1 | 93 | 16.6±3.8 | 63 | <0.001^**^ |
| Short K-KFAST at 12M | | 18.1±3.1 | 160 | 18.8±2.0 | 98 | 16.9±4.1 | 62 | <0.001^**^ |
| Short K-KFAST at 18M | | 18.3±2.9 | 158 | 18.7±2.8 | 99 | 17.7±2.9 | 59 | 0.02^*^ |
| Short K-KFAST at 24M | | 18.2±3.2 | 162 | 18.8±2.7 | 99 | 17.2±3.6 | 63 | 0.001^*^ |
| Short K-KFAST at 30M | | 18.0±3.3 | 168 | 18.9±2.1 | 99 | 16.6±4.1 | 69 | <0.001^**^ |
| Short K-KFAST at 36M | | 18.1±3.4 | 181 | 19.0±2.2 | 109 | 16.7±4.2 | 72 | <0.001^**^ |
| FIM at 3M | | 119.8±13.3 | 159 | 121.0±13.7 | 96 | 117.9±12.6 | 63 | 0.14 |
| FIM at 6M | | 121.1±10.7 | 158 | 121.8±11.1 | 95 | 120.0±10.1 | 63 | 0.31 |
| FIM at 12M | | 122.2±12.0 | 161 | 123.1±9.6 | 99 | 120.8±15.1 | 62 | 0.23 |
| FIM at 18M | | 122.4±12.1 | 160 | 123.5±9.6 | 100 | 120.6±15.3 | 60 | 0.14 |
| FIM at 24M | | 123.6±7.8 | 163 | 124.0±7.7 | 100 | 122.9±7.9 | 63 | 0.38 |
| FIM at 30M | | 122.7±10.7 | 168 | 123.8±7.6 | 99 | 121.1±13.9 | 69 | 0.10 |
| FIM at 36M | | 122.5±12.2 | 183 | 124.4±5.7 | 111 | 119.5±17.8 | 72 | 0.008^*^ |

FMA, Fugl-Meyer Assessment; FAC, Functional Ambulation Category; K-MMSE, Korean Mini-Mental State Examination; ASHA-NOMS, American Speech-Language-Hearing Association National Outcome Measurement System Swallowing Scale; Short K-FAST, Short version of the Korean Frenchay Aphasia Screening Test; FIM, Functional Independence Measure; D, days after stroke onset; M, months after stroke onset.

**p*<0.05, ***p*<0.001, compared between the young and old patients using independent t-test.
